# Prognostic Factors of Late-onset Hearing Loss in Infants With Congenital Cytomegalovirus and Normal Audiologic Assessment at Birth

**DOI:** 10.1097/INF.0000000000004960

**Published:** 2025-09-09

**Authors:** Danilo Buonsenso, Roberto Pedrero-Tomé, Francesco Raimondi, Serena Salomé, Vassiliki Papaevangelou, Garyfallia Syridou, María Ríos-Barnés, Clàudia Fortuny, Serena Villaverde, Joaquín de Vergas, Fernando Baquero-Artigao, Paula Rodríguez-Molino, Marie Antoinette Frick, Beatriz Álvarez-Vallejo, Jesús Saavedra-Lozano, Yves Fougere, Rut Del Valle, Fátima Ara-Montojo, Ina Foulon, Simona Mignogna, Oihana Muga Zuriarrain, Hermione Lyall, Isabel Vives-Oñós, Elena Colino, Olga Tsiatsiou, Elisenda Moliner, Francesca Garofoli, Irene Cuadrado, Daniel Blázquez-Gamero, on behalf of the European Registry of Children with Congenital CMV

**Affiliations:** From the * Department of Woman and Child Health and Public Health, Fondazione Policlinico Universitario A. Gemelli IRCCS, Rome, Italy; † Area Pediatrica, Dipartimento di Scienze della Vita e Sanità Pubblica, Università Cattolica del Sacro Cuore, Roma, Italia; ‡ Instituto de Investigación Hospital 12 de Octubre (imas12), Centro de Investigación Biomédica en Red en Enfermedades Infecciosas (CIBERINFEC), Madrid, Spain; § Division of Neonatology, Department of Translational Medical Sciences, University of Naples Federico II, Naples, Italy; ¶ Third Department of Pediatrics, National and Kapodistrian University of Athens, University General Hospital ATTIKON, Athens, Greece; ∥ Infectious Diseases Department, Hospital Sant Joan de Déu and Institut de Recerca Sant Joan de Déu, Barcelona, Spain. Centro de Investigación Biomédica en Red de Enfermedades Infecciosas (CIBERINFEC); ** Pediatric Infectious Diseases Unit, Hospital Universitario 12 de Octubre, Madrid, Spain; †† Otorhinolaryngology Department, Hospital Universitario 12 de Octubre, Madrid, Spain; ‡‡ Pediatrics, Infectious and Tropical Diseases Department, Hospital Universitario La Paz, Instituto Investigación Hospital La Paz (IDIPaz), Madrid, Spain, Centro de Investigación Biomédica en Red de Enfermedades Infecciosas (CIBERINFEC); §§ Pediatric Infectious Diseases and Immunodeficiencies Unit, Hospital Infantil. Vall d’Hebron Barcelona Hospital Campus. Barcelona, Catalonia, Spain; ¶¶ Pediatric Infectious Diseases Unit, Hospital Universitario Gregorio.Marañón, Universidad Complutense de Madrid. Madrid, Spain, Centro de Investigación Biomédica en Red en Enfermedades Infecciosas (CIBERINFEC); ∥∥ Unit of Pediatric Infectious Diseases and Vaccinology, Department of Woman Mother and Child, Lausanne University Hospital, Lausanne, Switzerland; *** Department of Pediatrics, Hospital Universitario Infanta Sofía, San Sebastián de los Reyes, Spain; ††† Hospital Universitario Puerta de Hierro, Spain; ‡‡‡ Department of Otolaryngology-Head and Neck Surgery, Universitair Ziekenhuis Brussel, Vrije Universiteit Brussel, Brussels, Belgium and De Poolster, Rehabilitation Centre, Brussels, Belgium; §§§ Catholic University of Rome, Medical School, Italy; ¶¶¶ Department of Pediatrics, Hospital Universitario Donostia, Donostia-San Sebastián, Spain; ∥∥∥ Paediatric Infectious Diseases, Imperial College Healthcare NHS Trust, and Imperial College, London, UK; **** Department of Pediatrics, Hospital Quironsalud Barcelona, Barcelona, Spain; †††† Department of Pediatrics, Complejo Hospitalario Universitario Insular-Materno Infantil de Las Palmas de Gran Canaria, Spain; ‡‡‡‡ 3rd Department of Pediatrics, Aristotle University of Thessaloniki and Hippokration General Hospital, Thessaloniki Greece; §§§§ Hospital de la Santa Creu i Sant Pau, Spain; ¶¶¶¶ SC Neonatologia e Terapia Intensiva Neonatale Fondazione IRCCS Policlinico S. Matteo, Pavia; ∥∥∥∥ Hospital Universitario de Getafe; ***** Universidad Complutense de Madrid, Spain.

**Keywords:** cytomegalovirus, congenital, late-onset hearing loss

## Abstract

**Background:**

Children with congenital cytomegalovirus (cCMV) can develop late-onset sensorineural hearing loss (LO-SNHL). In this study, we aim to assess the characteristics and predictors of LO-SNHL in infants with cCMV having normal hearing at the first neonatal assessment.

**Methods:**

A retrospective study within the European Registry of Children with cCMV (www.ccmvnet.org) was performed. Included children had cCMV and a normal first audiological assessment by Auditory Brainstem Response (ABR). Late-onset hearing loss (LO-SNHL) is defined as the presence of sensorineural hearing loss after an initial normal hearing test. Hearing evaluation was performed at birth, at 6 months of age, and at least annually up to 6 years of age.

**Results:**

Seven hundred twenty-one children with normal audiological tests at birth were included, and 47/721 (6.5%) developed LO-SNHL. LO-SNHL was diagnosed at a range of 4–65 months of age [median (IQR) age: 34.3 (15.1–48.7) months]. Children with LO-SNHL had a higher proportion of abnormalities on physical examination at birth (45.7% vs. 20.8%; *P* < 0.001): petechiae (17.4% vs. 6.0%; *P* = 0.008), splenomegaly (8.7% vs. 2.3%; *P* = 0.031), hepatomegaly (13% vs. 2.9%; *P* = 0.001), microcephaly (15.2% vs. 4.5%; *P* = 0.005) and small for gestational age (21.7% vs. 8.3% *P* = 0.005). Children with LO-SNHL showed lower platelet count at birth [177500.0 (88750.0–261250.0)/μL vs. 243500.0 (173000.0–304000.0)/μL; *P* = 0.012], and higher blood viral load at birth [3.7 log (3.3–4.4) vs. 3.4 log (2.7–3.9) IU/mL; *P* = 0.013] and had more frequent white matter involvement (27.7% vs. 14.7%; *P* = 0.03) and ventriculomegaly (20.7% vs. 4.6%; *P* = 0.001) on birth magnetic resonance imaging. Overall, symptomatic children at birth showed a higher risk of developing LO-SNHL than asymptomatic children (32/317, 10.1%, vs. 15/404, 3.7%; *P* < 0.0001). Among asymptomatic children, only 0.3% developed severe or profound LO-SNHL in the best ear.

In multivariate logistic regression analysis, ventriculomegaly [odds ratio (OR): 7.503 (1.78–27.9)], white matter abnormalities [OR: 3.19 (1.010–9.01)], and splenomegaly [OR: 3.679 (1.56–8.506)] at birth were associated with the development of LO-SNHL (Fig. 1).

**Conclusions:**

Among this large cohort of children with cCMV and a first normal audiological assessment, the risk of LO-SNHL was 6.5%. Asymptomatic children developed LO-SNHL in 3.7% of the cases versus 10.1% in symptomatic cases. In multivariate logistic regression analysis, ventriculomegaly, white matter abnormality, and splenomegaly at birth were associated with LO-SNHL.

Congenital cytomegalovirus (cCMV) is the commonest congenital infection in developed countries.^1^ cCMV infection can range from asymptomatic infection at birth to a severe, life-threatening disseminated disease.^2^ Although the time of maternal infection is of paramount importance in assessing the risk of long-term sequelae, in many cases, it is challenging to know the exact time of infection, or it is not possible (nonprimary infection). cCMV is considered the most frequent cause of nongenetic sensorineural hearing loss (SNHL) in childhood.^3,4^ It is estimated that about 25% of cases of SNHL in children up to 4 years of age are attributable to cCMV.^4^ Infants with symptomatic infections at birth have a higher risk of developing late onset SNHL (LO-SNHL) (up to one-third), but also asymptomatic newborns can develop it during childhood, and most cases occur before 3 years of age.^5,6^ However, available evidence is mostly based on relatively small cohorts that also include newborns with abnormal hearing at birth and do not provide serial follow-up at specific time points up to 3–5 years of age, the period recommended for follow-up by most guidelines.^7^

Knowing prognostic factors of the development of LO-SNHL may have practical consequences, as children at high risk of LO-SNHL could be candidates for antiviral treatment. While clinical trials conducted in symptomatic infants showed that antiviral treatment with ganciclovir/valganciclovir may prevent hearing deterioration,^8,9^ the evidence about antiviral treatment in asymptomatic or mildly symptomatic cCMV cases is still lacking.^7^ Therefore, therapeutic practices widely differ worldwide according to local guidance and expert opinions.^10,11^ In addition, there are few studies that have addressed risk factors for the development of LO-SNHL in children with normal hearing at birth, with no clear link with image findings at birth, but with an association with the first trimester of maternal infection.^12^ Given current gaps, we performed this study to assess the burden of LO-SNHL and its prognostic factors in a large cohort of European infants enrolled in the cCMVnet Registry, who had a normal audiological evaluation at birth and a documented hearing follow-up of at least 6 months.

## METHODS

### Study Population

This retrospective study was performed within the European Registry of Children with Congenital Cytomegalovirus Infection (cCMVnet registry; www.ccmvnet.org).^13^ This is a European Registry of children with cCMV and includes 1461 children followed in 57 hospitals from 8 countries (Spain, Greece, Italy, United Kingdom, Portugal, Belgium, Czech Republic and Switzerland). This registry includes patients born after 2011 and until 2024, and prospectively followed up, enrolled from national and unicentric cohorts.

Children with confirmed cCMV infection (positive polymerase chain reaction and/or urine viral culture for CMV in the first 21 days of life), followed in participating centers, were offered to be included in the registry. Anonymized clinical information was stored using the REDCap data collection system.^11^ REDCap is hosted in a secure online server at the Instituto de Investigación Hospital 12 de Octubre (imas12). cCMVnet Registry has been approved by the reference Ethics Committee in Hospital Universitario 12 de Octubre (IRB number: 20/495) and the IRB of each participating center. Parents or legal guardians signed informed consent at the inclusion in the registry.

Demographic and patient data, type of maternal infection, type of neonatal infection (symptoms at physical examination), blood count and liver enzymes at birth and viral load in blood and urine were recorded. Imaging results at birth [cranial ultrasound (cUS) and/or cranial magnetic resonance imaging (MRI)] performed in the first 28 days of life, and ophthalmologic findings were also included. Image findings in cUS and MRI potentially related to cCMV and recorded in the study were white matter abnormalities (WMAs), lenticulostriate vasculopathy, calcifications, periventricular cysts, ventriculomegaly, caudothalamic and subependymal cysts and/or germinolysis, polymicrogyria, lissencephaly and other migrational abnormalities and ventricular septation.

### Hearing Evaluation

Hearing screening is universally performed on all newborns in participating centers by automated auditory brainstem response (A-ABR) or otoacoustic emissions. If hearing screening fails in any ear, it is repeated in 2–6 weeks.

After birth, all children received audiologic examination by an ear, nose and throat specialist, and auditory brainstem response (ABR), tympanometry and microscopic ear evaluation were performed. In some centers, auditory steady-state response is performed when hearing loss is suspected to quantify the degree of hearing loss.

Children with normal hearing are evaluated with ABR and/or behavioral audiometry at 6 months of age and then yearly for up to 5 years of age. SNHL was defined as the presence of hearing thresholds of >30 dBnHL in ABR in any ear or if pure-tone average is above a threshold of 20 dBHL in any ear.

Quantification of the degree of SNHL was performed according to the classification of Bureau International d’Audiophonologie (BIAP 1996), being mild hearing loss pure-tone average between 21 and 40, moderate 41–70, severe 71–90, profound >90 dB.^14,15^

### Other Definitions

cCMV: was defined as the presence of a positive CMV-polymerase chain reaction in urine, blood, or cerebrospinal fluid or CMV isolation in urine cultures in the first 21 days of life.^16^

Small for gestational age: weight at birth with Z-score <−2, adjusted by sex and gestational age, and according to the reference charts.^17^

Microcephaly: head circumference Z-score <−2 according to the reference charts adjusted by sex and gestational age.^18^

Symptomatic cCMV: Infants were categorized as “symptomatic” if they present any of the following signs and symptoms: abnormal physical examination at birth [petechiae, splenomegaly, hepatomegaly, jaundice, hypotonia, lethargy, seizures, poor sucking reflexes, motor paresis, microcephaly (head circumference <−2 Z-score head circumference)], small for gestational age (<−2 Z-score head circumference), abnormal blood test (thrombocytopenia <100,000 and/or alanine aminotransferase ≥80 UI/dL), chorioretinitis, SNHL, abnormalities in imaging (WMA), ventricular adhesions, intracranial calcifications, ventriculomegaly, cerebellar hypoplasia, periventricular cysts, caudothalamic/subependymal cysts, hydrocephalus, lissencephaly, polymicrogyria and other migration abnormalities. Children with isolated lenticulostriate vasculopathy were not included in the “symptomatic” group.^7,19^

Best ear was referred to the ear with the best hearing result; for example, if you have moderate (left ear) severe (right ear), you have moderate SNHL in the best ear.

### Statistical Analysis

To elucidate the characteristics of the study cohort, pertinent variables were systematically summarized and presented in tabular form using the compare Groups R package.^20^ Categorical attributes were presented as frequency distributions, and comparative analyses between different groups were conducted using Fisher exact test and the χ^2^ test. For continuous variables, due to the non-normal distribution of the variables analyzed in the proposed groups, the Mann–Whitney *U* test was employed. These continuous attributes were represented in terms of their medians and interquartile ranges (IQR). Additionally, a survival analysis was conducted using the Kaplan–Meier method to visualize the follow-up times at which new cases of deafness occurred. The choice of the Mann–Whitney test was based on the need to compare 2 groups (infants with and without hearing loss) in the presence of a non-normal distribution of the variable analyzed, which was verified beforehand. Since no comparisons between more than 2 groups are addressed in this study, the use of ANOVA—a parametric test designed for such comparisons—was not considered appropriate.

To conclude, a multivariate logistic regression model was developed to predict the development of new LO-SNHL. The predictor variables of the final model were subjected to collinearity tests using the variance inflation factor. The final model was evaluated using McFadden’s index.

## RESULTS

From January 2020 to December 2024, 1461 patients with cCMV have been included in the registry, and 721 patients with normal neonatal audiological screening met the study criteria and were included (404 with asymptomatic and 317 with symptomatic cCMV infection). In total, 47/721 (6.5%) children with initial normal audiological screening developed LO-SNHL during a median follow-up of 30.7 (14.4–47.9) months. LO-SNHL was diagnosed at a median of 34.3 months (IQR: 15.1–48.7); range of 4–65 months and more than 95.8% before the age of 5 years. In the last evaluation, 27/47 (57.4%) had unilateral LO-SNHL and 20/47 (42.6%) had bilateral LO-SNHL. Among 20 kids with bilateral LO-SNH, hearing loss in best ear was mild (21–40 dB) in 11 (23.4%) cases, moderate (41–70 dB) in 6 (12.8%), severe (71–90 dB) in 2 (4.3%) and profound (>90 dB) in 1 child (2.1%). Risk of developing bilateral LO-SNHL is 2.8% (20/721), and risk of moderate-severe-profound LO-SNHL in the best ear is 1.2%. Two kids (2/721; 0.3%) with LO-SNHL required cochlear implants.

Details about demographic and clinical findings of newborns and mothers are reported in Table 1 and Table, Supplemental Digital Content 1, https://links.lww.com/INF/G345, respectively. Data about microbiology and laboratory findings at birth are listed in Table 2. In Table 3, imaging findings are listed.

**TABLE 1.** Clinical Data of Newborns with cCMV Infection That Developed or Not LO-SNHL

	No LO-SNHL (N = 674)	Yes LO-SNHL (N = 47)	*P*
Age at diagnosis (days) [median (IQR)]	2.0 (0.0–6.0)	2.0 (0.0–5.0)	0.729
Age (mother) years [median (IQR)]	33.0 (28.0–36.0)	33.0 (30.5–35.0)	0.626
Gestational age at delivery (weeks) [median (IQR)]	38.0 (37.0–39.0)	38.0 (38.0–39.0)	0.393
Gestational age week <37 (%)	140 (21.1)	7 (14.9)	0.403
Birth weight (gr) [median (IQR)]	2920.0 (2400.0–3340.0)	2660.0 (2285.0–3152.5)	0.184
Z-Score newborn weight [median (IQR)]	−0.3 (−1.3 to 0.6)	−0.8 (−1.8 to 0.2)	**0.023**
Birth weight category (%)			
≥1500 g	620 (93.9)	45 (95.7)	0.852
<1500 g	40 (6.1)	2 (4.3)	
Birth height (cm) [median (IQR)]	48.5 (46.0–50.0)	48.0 (46.0–49.5)	0.256
Z-Score newborn height [median (IQR)]	0.0 (−1.2 to 0.7)	−0.8 (−1.9 to 0.1)	**0.042**
Birth head circumference (cm) (median (IQR))	33.5 (32.0–35.0)	33.0 (32.0–34.0)	0.113
Z-Score Newborn head circumference (median (IQR))	−0.6 (−2.0 to 0.5)	−1.6 (−2.5 to 0.0)	**0.037**
Small for gestational age (SGA) (<−2 Z-Score or ≤ 2nd centile) (%)	55 (8.3)	10 (21.7)	**0.005**
Asymptomatic cCMV infection (n = 404)	389 (96.3)	15 (3.7)	**<0.001**
Symptomatic cCMV infection (n = 317)	285 (89.9)	32 (10.1)	
Isolated SGA	7 (1.0)	0 (0.0)	**1.000**
Abnormal physical examination at birth (%)	138 (20.8)	21 (45.7)	**<0.001**
Microcephaly (<−2 Z-Score or ≤2nd centile) (%)	30 (4.5)	7 (15.2)	**0.005**
Seizures (%)	0 (0.0)	0 (0.0)	--
Splenomegaly (%)	15 (2.3)	4 (8.7)	**0.032**
Hepatomegaly (%)	19 (2.9)	6 (13.0)	**0.001**
Hypotonia (%)	14 (2.1)	2 (4.3)	0.634
Jaundice (%)	27 (4.1)	3 (6.5)	0.673
Petechiae/purpura (%)	40 (6.0)	8 (17.4)	**0.008**
Chorioretinis (%)	4 (0.6)	2 (4.3)	0.069
Last visit–follow-up			
Age (months) [median (IQR)]	27.6 (13.5–44.5)	30.7 (14.4–47.9)	0.825
Motor impairment (paresis spasticity) (%)	39 (5.9)	8 (17)	**0.006**
Epilepsy (in treatment) (%)	9 (1.4)	2 (4.3)	0.311
Visual impairment (%)	10 (1.5)	2 (4.3)	0.364
Any sequelae (%)	42 (6.5)	47 (100.0)	<0.001

Bold values indicates statistically significant *P* values.

**TABLE 2.** Main Laboratory and Microbiological Data of Newborns with cCMV Infection That Developed or Not LO-SNHL

	No LO-SNHL (N = 674)	Yes LO-SNHL (N = 47)	*P*
Blood test at birth (first week of life) (%)			
Normal	453 (85.3)	25 (64.1)	**0.001**
Abnormal	78 (14.7)	14 (35.9)	
Newborn: ALT (GPT) (U/L) [median (IQR)]	18.0 (12.0–27.0)	20.0 (14.0–31.0)	0.170
Newborn: AST (GOT) (U/L) [median (IQR)]	45.0 (31.0–62.0)	54.0 (39.5–70.0)	0.084
Newborn: direct bilirubin (mg/dL) [median (IQR)]	0.6 (0.4–2.0)	1.1 (0.4–5.0)	0.329
Newborn: hemoglobin (g/dL) [median (IQR)]	16.0 (14.2–18.0)	16.1 (15.3–18.1)	0.336
Newborn: platelets (cs/mm^3^) [median (IQR)]	243500.0 (173000.0–304000.0)	177500.0 (88750.0–261250.0)	**0.012**
Newborn: leucocytes (cs/mm^3^) [median (IQR)]	11610.0 (8900.0–15205.0)	13050.0 (11207.5–16850.0)	0.178
Newborn: lymphocytes (cs/mm^3^) [median (IQR)]	5047.0 (4050.0–6970.0)	7000.0 (4793.5–7500.0)	0.109
Newborn: neutrophils (cs/mm^3^) [median (IQR)]	3940.0 (2040.0–7870.0)	4450.0 (2715.0–7330.0)	0.667
Lumbar puncture performed (%)			
Yes	209 (35.5)	19 (51.4)	0.133
Newborn: leucocytes in CSF [median (IQR)]	15.0 (6.0–30.0)	14.5 (10.0–23.5)	0.977
Newborn: –in CSF (mg/dL) [median (IQR)]	100.0 (74.9–137.0)	90.0 (72.0–112.3)	0.414
Amniocentesis: CMV-PCR (%)			
Positive	107 (66.0)	9 (100.0)	0.079
Negative	55 (34.0)	0 (0.0)	
Amniocentesis: CMV viral load [median (IQR)]	199000.0 (8650.0–2147500.0)	115000.0 (11554.0–2030000.0)	0.976
Child: urine CMV viral load [median (IQR)]	1748949.0 (93935.0–10000000.0)	10000000.0 (177000.0–29999999.0)	0.142
Blood CMV-PCR (%)			
Positive	443 (81.0)	33 (89.2)	0.305
Negative	104 (19.0)	4 (10.8)	
Child: blood CMV viral load (log) [median (IQR)]	3.4 (2.7–3.9)	3.7 (3.3–4.4)	**0.013**
CSF CMV-PCR (%)			
Positive	35 (14.8)	7 (26.9)	0.189
Negative	201 (85.2)	19 (73.1)	
Child: CSF CMV viral load [median (IQR)]	581.0 (150.0–1482.8)	819.0 (288.8–1602.2)	0.756

ALT, alanine aminotransferase; AST, aspartate aminotransferase; CSF, cerebrospinal fluid; GOT, glutamate pyruvate transaminase; GPT, glutamate oxaloacetate transaminase.

**TABLE 3.** Main Imaging Data of Newborns with cCMV Infection That Developed or Not LO-SNHL

	No LO-SNHL (N = 674)	Yes LO-SNHL (N = 47)	*P*
Child first cranial ultrasound (%)			
Normal	432 (68.4)	24 (55.8)	0.126
Abnormal	200 (31.6)	19 (44.2)	
Abnormal without lenticulostriate vasculopathy	152 (24.1)	16 (37.2)	0.080
White matter abnormalities (focal/multifocal) (%)	13 (2.1)	2 (4.7)	0.560
White matter abnormalities (Diffuse) (%)	7 (1.1)	0 (0.0)	1.000
Ventriculomegaly	21 (3.3)	1 (2.3)	1.000
Ventricular adhesions/septations (%)	3 (0.5)	0 (0.0)	1.000
Lenticulostriate vasculopathy (%)	84 (13.3)	8 (18.6)	0.451
Periventricular cysts (%)	37 (5.9)	4 (9.3)	0.558
Caudothalamic/subependymal cysts/germinolysis (%)	51 (8.1)	5 (11.6)	0.594
Calcifications (%)	37 (5.5)	6 (12.8)	0.086
Child first cranial MRI (%)			
Normal	261 (63.7)	9 (31.0)	**0.001**
Abnormal	149 (36.3)	20 (69.0)	
White matter abnormalities (focal/multifocal) (%)	65 (15.9)	12 (41.4)	**0.001**
White matter abnormalities (diffuse) (%)	34 (8.3)	1 (3.4)	0.565
Isolated white matter abnormalities without any other abnormality	51 (7.6)	5 (10.6)	0.555
Ventricular adhesions/septations (%)	7 (1.7)	0 (0.0)	1.000
Lenticulostriate vasculopathy (%)	4 (1.0)	1 (3.4)	0.759
Calcifications (%)	22 (5.4)	4 (13.8)	0.083
Enlargement of the cisterna magna (%)	2 (0.5)	2 (6.9)	**0.012**
Ventriculomegaly (%)	19 (4.6)	6 (20.7)	**0.001**
Cerebellar hypoplasia (%)	6 (1.5)	1 (3.4)	0.954
Periventricular cysts (%)	24 (5.9)	4 (13.8)	0.194
Lissencephaly/other migrational abnormalities (%)	5 (1.2)	1 (3.4)	0.864
Caudothalamic/subependymal cysts/germinolysis (%)	30 (7.3)	2 (6.9)	1.000
Polymicrogyria (%)	5 (1.2)	1 (3.4)	0.864

Symptomatic children at birth showed a higher risk of developing LO-SNHL than asymptomatic children (32/317, 10.1%, vs. 15/404, 3.7%; *P* < 0.0001) (Table 1). Children with asymptomatic disease at birth showed no LO-SNHL in the best ear in 98.5% of the cases, versus 92.5% of the symptomatic (*P* = 0.001). Asymptomatic children only developed severe/profound LO-SNHL in the best ear in 1 case (0.3%) versus 0.7% of the symptomatic (Table 4).

**TABLE 4.** LO-SNHL Status by Ear and Presence of Symptoms at Birth

	Level	Asymptomatic	Symptomatic	*P*	No LO-SNHL	LO-SNHL	*P*
n		404	317		674	47	
Last visit LO-SNHL in any ear							
Last visit—hearing-loss right (%)	No hearing loss	379 (97.4)	272 (89.2)	<0.001	674 (100.0)	14 (29.8)	<0.001
	Yes, mild: 21–40 dB	7 (1.8)	15 (4.9)		0 (0.0)	14 (29.8)	
	Yes, moderate: 41–70 dB	1 (0.3)	14 (4.6)		0 (0.0)	13 (27.7)	
	Yes, severe: 71–90	1 (0.3)	2 (0.7)		0 (0.0)	3 (6.4)	
	Yes, profound: > 90 dB	1 (0.3)	2 (0.7)		0 (0.0)	3 (6.4)	
Last visit—hearing-loss left (%)	No hearing loss	381 (97.4)	274 (89.8)	0,001	674 (100.0)	18 (38.3)	<0.001
	Yes, mild: 21–40 dB	5 (1.3)	14 (4.6)		0 (0.0)	11 (23.4)	
	Yes, moderate: 41–70 dB	3 (0.8)	11 (3.6)		0 (0.0)	10 (21.3)	
	Yes, severe: 71–90	2 (0.5)	4 (1.3)		0 (0.0)	6 (12.8)	
	Yes, profound: > 90 dB	0 (0.0)	2 (0.7)		0 (0.0)	2 (4.3)	
LO-SNHL in last visit in any ear		15 (3.7)	32 (10.1)	0.001	0 (0.0)	47 (100.0)	<0.001
Last visit—hearing-loss in best ear (%)	No hearing loss	383 (98.5)	282 (92.5)	0.001	674 (100.0)	27 (57.4)	<0.001
	Yes, mild: 21–40 dB	4 (1.2)	7 (2.1)		0 (0.0)	11 (23.4)	
	Yes, moderate: 41–70 dB	0 (0.0)	6 (1.8)		0 (0.0)	6 (12.8)	
	Yes, severe: 71–90 dB	1 (0.3)	1 (0.3)		0 (0.0)	2 (4.3)	
	Yes, profound >90 dB	0 (0.0)	1 (0.3)		0 (0.0)	1 (2.1)	
Last visit bilateral LO-SNHL		5 (1.2)	15 (4.9)	0.001	0 (0.0)	20 (42.6)	<0.001

### Univariate Analysis According to the Development of LO-SNHL

Children who developed LO-SNHL had lower Z-score at birth for weight [−0.8 (−1.8 to 0.2) vs. −0.3 (−1.3 to 0.6); *P* = 0.023], height [−0.8 (−1.9 to 0.1) vs. 0.0 (−1.2 to 0.7); *P* = 0.042] and head circumference [−1.6 (−2.5 to 0.0) vs. −0.6 (−2.0 to 0.5); *P* = 0.037). Children small for gestational age (< −2 Z-score birth weight for gestational age) had a higher risk for LO-SNHL; however, none of the children with isolated small for gestational age (SGA) (SGA without other symptoms) developed LO-SNHL. Children with LO-SNHL had a higher proportion of abnormalities in physical examination at birth (45.7% vs. 20.8%; *P* < 0.001): petechiae (17.4% vs. 6.0%; *P* = 0.008), splenomegaly (8.7% vs. 2.3%; *P* = 0.031), hepatomegaly (13% vs. 2.9%; *P* = 0.001), microcephaly (15.2% vs. 4.5%; *P* = 0.005) and chorioretinitis (0.6% vs. 4.3%; *P* = 0.069) (Tables 1–3).

Children with LO-SNHL showed lower platelet count at birth [177,500 (88,750.0–261,250.0)/μL vs. 243,500 (173,000–304,000)/μL; *P* = 0.012] and higher blood viral load at birth [3.7 log (3.3–4.4) vs. 3.4 log (2.7–3.9) IU/mL; *P* = 0.013] (Table 2).

Abnormal US at birth was not associated with LO-SNHL, but children with LO-SNHL showed a higher proportion of abnormalities in cranial MRI (69% vs. 36.3%; *P* = 0.001). WMAs and ventriculomegaly were more frequent in children who developed LO-SNHL (41.4% vs. 15.9%; *P* = 0.001, and 20.7% vs. 4.6%; *P* = 0.001, respectively).

In the last visit available, children with LO-SNHL showed a higher rate of microcephaly (11.9 % vs. 3.9%), more motor problems (17.8% vs. 5.9%) and overall sequelae rate was higher (100% vs 6.5%) than in children without LO-SNHL.

### Among Children with LO-SNHL 63.8% (30/47) Received Antiviral Treatment

Thirty-four percent (16/47) received valganciclovir and 29.8% (14/47) received combined treatment (valganciclovir and ganciclovir). In infants without LO-SNHL, 39% (263/674) received antiviral treatment: 25.5% (170/674) received valganciclovir and 13.8% (93/674) received ganciclovir plus valganciclovir. There were no clear significant differences in LO-SNHL among treated and untreated patients (*P* = 0.057).

A detailed assessment of LO-SNHL by ear and symptomatic status at birth of newborns with cCMV is given in Table 4, showing that newborns with symptomatic infection have a higher risk of developing LO-SNHL in either ear, but also a more profound hearing loss.

**FIGURE 1.**
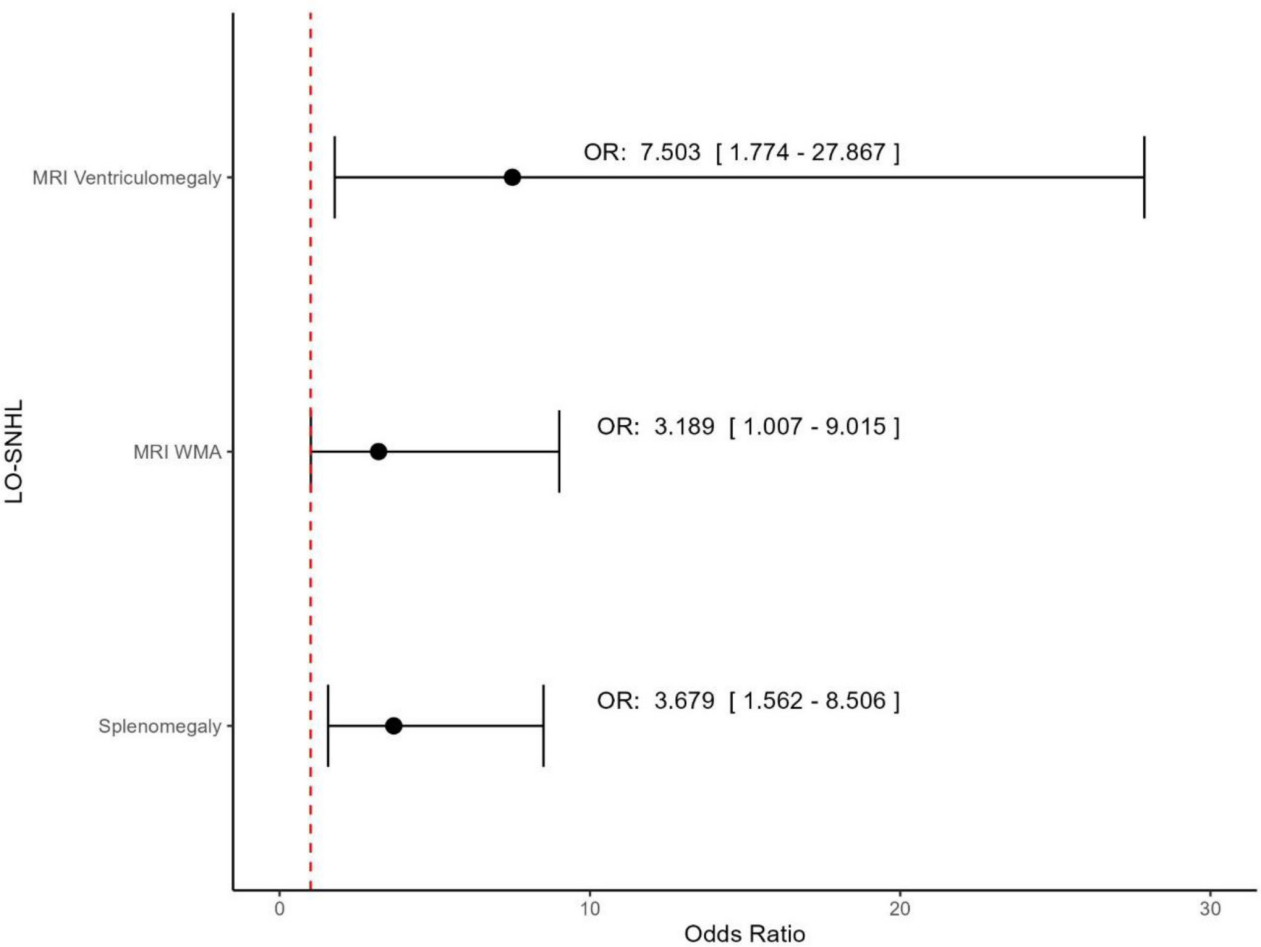
Multivariate logistic regression analysis for the outcome of late-onset SNHL.

In multivariate logistic regression analysis, ventriculomegaly on MRI [OR: 7.503 (1.77–27.86)], WMAs at MRI [OR: 3.18 (1.00–9.01)] and splenomegaly [OR: 3.679 (1.56–8.506)] were associated with the development of LO-SNHL (Fig. 1).

**FIGURE 2.**
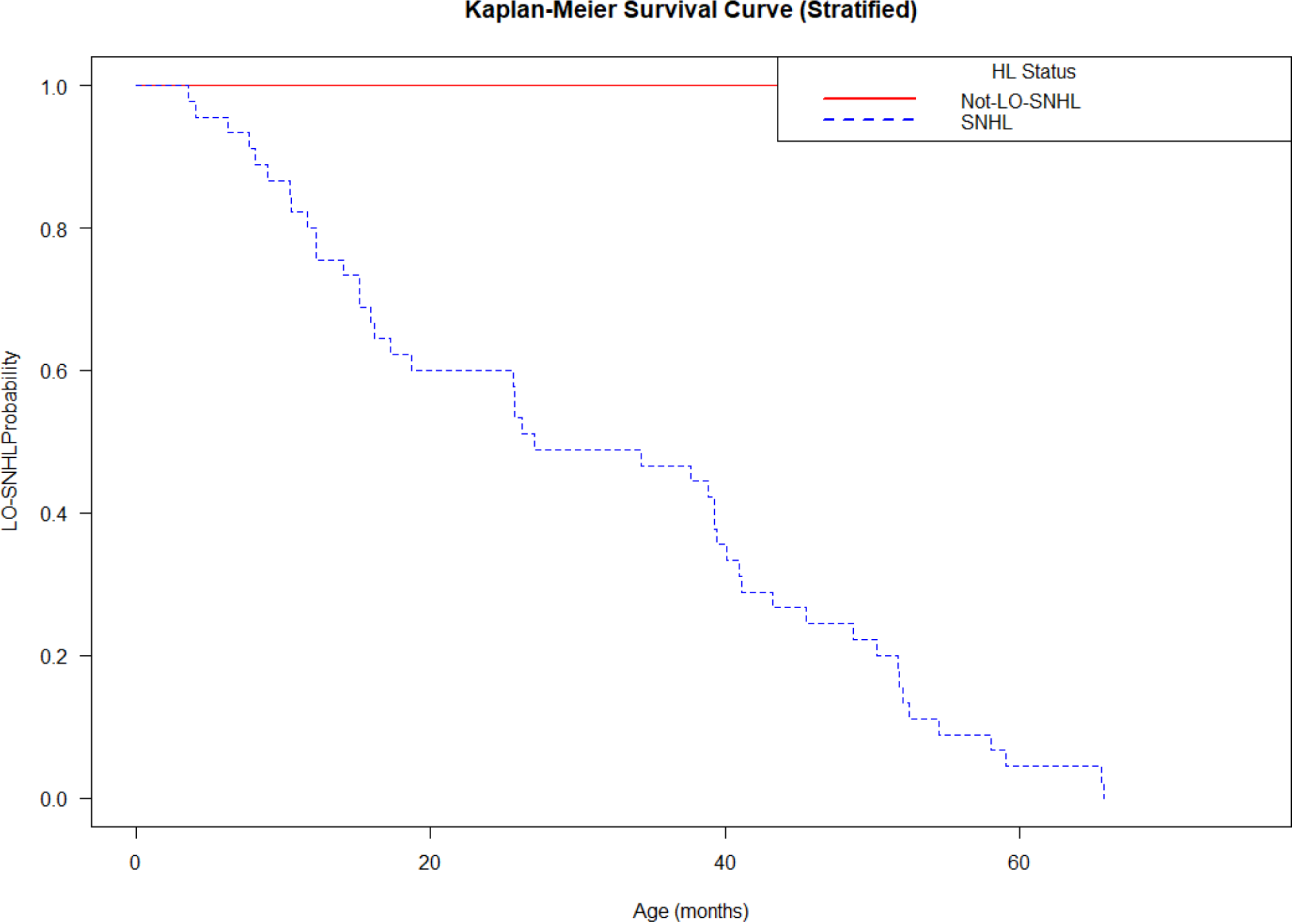
Time series analysis for the outcome of late-onset SNHL.

Figure 2 and Table, Supplemental Digital Content 2, https://links.lww.com/INF/G346 show the timing for development of LO-SNHL in the cohort. The timing for the detection of the outcome is variable; however, almost all cases were detected within the first 65 months of life.

## DISCUSSION

This is the largest multicentric study to assess LO-SNHL in newborns with cCMV infection who had a normal initial audiological assessment at birth. In our cohort, 47/721 (6.5%) children with initial normal audiology developed LO-SNHL. Symptomatic infants had a higher risk of LO-SNHL (10.1% vs. 3.7%; *P* < 0.0001).

We found that splenomegaly and image abnormalities like focal/multifocal WMA and ventriculomegaly were associated with LO-SNHL. Other findings, like isolated SGA, were not associated with LO-SNHL.

In a recent study from the Flemish registry, they found a similar proportion of LO-SNHL in symptomatic children (11.8%; 9/76) versus asymptomatic children (12.1%; 30/247).^21^

A report from 2014 suggested that 18.1% of symptomatic children had a delayed onset of hearing loss.^5^ According to Goderis et al^5,6^, LO-SNHL occurred in 10.6% of symptomatic and in 7.8% of asymptomatic infants with cCMV. This data is like the results in our study. However, the authors mention that previous hearing impairment in one ear was a risk factor for the development of LO-SNHL in the contralateral ear, suggesting that some of these children may not have had an initial entirely normal hearing assessment, while in the present study, the definition of LO-SNHL was more rigorous.

Foulon et al^22^ prospectively evaluated hearing loss in children with proven cCMV. In this cohort, among the 157 children with normal hearing thresholds at the first visit, 7 developed late-onset hearing loss (4.5%). However, 3 of them had other possible factors that might have contributed to the risk of SNHL, and the risk of LO-SNHL was even lower (2.5%) if those cases were excluded. In line with our findings, SNHL was more frequent in symptomatic children. The study by Foulon et al^22^ had a high proportion of asymptomatic children, and the risk of LO-SNHL is like what has been found in our study. Moreover, the risk of severe or profound LO-SNHL in asymptomatic children is low.

The risk of LO-SNHL in asymptomatic children is low in our study (3.7%), and only 0.3% of children developed severe or profound SNHL in the best ear. This low risk of LO-SNHL in asymptomatic children is an important message for parents because this possibility generates important anxiety among parents during the follow-up. Moreover, only 2 children in this group required cochlear implants.

In another cohort in China, including 141 children with asymptomatic cCMV infection, LO-SNHL was found in 4 cases (2.8%).^23^ Interestingly, in this cohort, no maternal or child factors, except saliva viral load at birth, were associated with an increased risk of developing LO-SNHL. We did not find differences in urine viral load (saliva was not analyzed); however, a higher blood viral load was found in children with LO-SNHL. Although in different studies the risk of LO-SNHL seems to increase with higher viral loads in different fluids, we could not establish a cutoff for high or low risk of developing LO-SNHL.

Two Italian studies have also attempted to document the burden of LO-SNHL. In an Italian cohort of 103 children from 2 centers, 28 had a hearing impairment, and 4 had LO-SNHL. However, no details are provided on the timing of diagnosis and how the first hearing evaluation was performed.^24^ In another center in Southern Italy, in a cohort of 102 purely asymptomatic cCMV-infected infants, no one developed LO-SNHL after a mean follow-up period of 3.3 years, with a median of 2.8 years.^3^

An important finding of our study is that WMA abnormalities detected on MRI were associated with a higher risk of LO-SNHL, reinforcing the latest studies suggesting a potential prognostic role of MRI in children with cCMV infection. A recent retrospective, single-center observational study, including 225 patients with cCMV who had a neonatal brain MRI with diffusion-weighted imaging, found that the white matter apparent diffusion coefficient was significantly higher in patients with neonatal hearing loss and cognitive and motor impairment (*P* < 0.05).^25^ In another study, 2 pediatric radiologists, blinded to clinical data, independently scored the white matter in 286 newborns with congenital CMV. Abnormal white matter was associated with neonatal hearing loss and lower motor scores, with a tendency towards impaired cognitive development.^26^ SNHL was also more frequent in newborns with MRI brain abnormalities in a smaller study (36 patients, 11 of them with MRI abnormalities and SNHL).^27^ In another study of 160 infants with cCMV (103 symptomatic), temporal-pole WMAs, and not the extent of WMAs, were associated with moderate/severe disability (OR: 7.8; 1.4–42.8), specifically severe SNHL or SNHL combined with other moderate/severe disabilities (OR: 16.2; 1.8–144.9).^28^ However, a study published by Craeghs et al^13^ found an association between hearing loss and image finding in cUS and MRI, but could not establish this association in LO-SNHL.^12^ In a series of 17 patients in the United States, the majority (59%) of infants whose cCMV infections were identified after failing newborn hearing screening had abnormal brain MRIs.^29^ Conversely, a small study from the Republic of Korea, including 31 patients, found no association between MRI findings and SNHL.^30^ However, none of these studies were focused on the subgroup of children without SNHL at birth, and the sample size was lower than in our study.

Although 69% of children with LO-SNHL had abnormalities on MRI, 31% of children with normal imaging also developed LO-SNHL. Additionally, 36.3% of children with abnormalities on MRI did not develop LO-SNHL. None of the treatment studies for cCMV undertook routine MRI imaging at baseline, so there are currently no correlations between treatment outcomes and baseline imaging. Altogether, these findings suggest that we need further evidence to introduce MRI as a routine diagnostic test to all children with asymptomatic cCMV infection, as the test is expensive and would not be sustainable at every center. So far, the ultrasound remains a primary indication, as it is radiation-free and accessible worldwide.

The mechanism of LO-SNHL remains poorly understood. Recent studies suggest that this may involve immune responses, the release of inflammatory factors by natural killer (NK) cells, apoptosis of cochlear spiral ganglion cells, and potential changes due to vascular dysfunction.^31^ A recent study discovered biosignatures of infants with symptomatic and asymptomatic cCMV are indistinguishable, both showing that the immune responses of infants with asymptomatic and symptomatic cCMV infection are not different. Random forest analyses of initial samples in infants with cCMV, irrespective of their clinical classification, identify a 16-gene classifier signature associated with the development of SNHL with 92% accuracy, suggesting its potential value as a biomarker.^32^ These data reinforce our findings that even asymptomatic children at birth can develop LO-SNHL, possibly due to chronic inflammation.

In our series, most cases (93.5%) of LO-SNHL are diagnosed before the fifth year of life. These findings are in line with previous reports suggesting that hearing loss occurs throughout the first several years of life and most cases are diagnosed before 5 years of age, although usually much earlier.^22,33–36^ Average onset of LO-SNHL is earlier in symptomatic children than in asymptomatic children.^12^

Also, our findings reinforce the association between cCMV infection and negative neurological outcomes.^37^ While any sort of neurologic and behavioral problems were diagnosed in cCMV patients independently from audiologic status, those with LO-SNHL had a statistically significantly higher risk of motor impairment at the last visit.

As advised by the American Academy of Audiology^38^ and the European Congenital Infection Initiative,^39^ our findings on LO-SNHL support the recommendations for close audiological follow-up of children with cCMV during the first 5–6 years of life, including children with normal hearing at birth, despite, as we have shown, the risk of LO-SNHL is much less in children who do not have symptoms at birth (3.7% in clinically asymptomatic vs. 10.1% in symptomatic newborns). Children with cCMV and SNHL and/or neurological consequences should have follow-up with: developmental pediatrics, infectious diseases, neurology, ophthalmology, speech-language pathology, physical therapy, pediatric otolaryngology, and parent–family support.^40^

Of note, we found that children with LO-SNHL showed a higher blood viral load at birth (3.7 log (3.3–4.4) vs. 3.4 log (2.7–3.9) IU/mL; *P* = 0.013). However, this data was not confirmed at the multivariate analyses, suggesting that blood viral load may not be a primary factor in defining the risk of subsequent LO-SNHL. This point would be in line with a previous large study showing that peripheral blood viral load is not associated with hearing loss in children with congenital CMV infection, although a viral load of <3500 ge/mL was associated with a lower risk of hearing loss in children born with asymptomatic congenital infection.^41^

Our study has limitations: despite having been performed in a prospective cohort, this is a retrospective study. Although this is the largest European cohort of children with CMV infection, it is difficult to recruit many children with LO-SNHL. Most asymptomatic patients in our cohort were identified through maternal screening programs conducted in specific participating centers (Spain, Greece, Italy and Belgium). It is important to note that congenital CMV infections resulting from primary maternal infection during the first trimester are associated with a higher risk of long-term sequelae, even in infants who are asymptomatic at birth. This factor may have contributed to overestimating the risk of LO-SNHL in our study population. Given the multicenter nature of the study, some heterogeneity existed in the timing and frequency of audiological follow-up, which may have influenced the precise identification of the onset of hearing loss. However, in the cCMVnet cohort, at least 1 evaluation of hearing was performed at 6 months of age in all children, and after 12 months of age at least once yearly. On a similar note, from the standpoint of representativeness and the applicability of results, working with samples from various centers undoubtedly adds value. However, we acknowledge that, regarding the assessment of viral load, the absence of a centralized laboratory for sample processing represents a limitation. In addition, audiological testing is extremely complex in young infants for multiple reasons, and this can potentially impact the results. The timing of maternal infection has not been addressed in this study and is one of the most relevant prognostic factors for SNHL,^12,38^ and WMA, hepatomegaly, symptomatic infection may well be an expression of a first-trimester infection and reflect the higher risk of LO-SNHL. Another limitation is that MRI has not been performed in all children. Lastly, maternal treatment with valaciclovir has been recently introduced in most centers. As in this retrospective cohort, we do not have mothers who received valaciclovir in pregnancy, our future studies will hopefully inform if this treatment benefits long-term audiological and neurocognitive outcomes. We have not assessed in depth the role of antiviral treatment in newborns on LO-SNHL, as decisions of treatments may be based on neonatal symptoms, and associations with the audiological outcomes may be biased.

In conclusion, our study showed that among a large cohort of newborns with cCMV infection with a first normal audiological assessment, overall, a low percentage of children developed LO-SNHL. Indeed, of those who were asymptomatic at birth, only 3.7% developed LO-SNHL. Among symptomatic children, those with splenomegaly and image abnormalities (WMA and ventriculomegaly) at birth have a higher risk of developing LO-SNHL.

## ACKNOWLEDGMENTS

We acknowledge families and patients for their invaluable support and collaboration in the *European Registry of Children with Congenital CMV (cCMVnet registry).*

cCMVnet registry study group: Hospital Arnau de Vilanova Lleida: Xavier Bringue Espuny. Hospital Barbanza A Coruña: Paula Sánchez Pintos. Hospital Cáceres: Elena María Márquez Isidro, María Jesús García García. Hospital Clínico San Carlos: Araceli Corredera Sánchez, Marta Illán. Hospital de Alcorcón: Antonio Cuñarro. Hospital de la Axarquía: Antonio Francisco Medina Claros. Hospital de la Santa Creu i Sant Pau: Elisenda Moliner Calderón. Hospital de Málaga: Begoña Carazo Gallego, Esmeralda Núñez Cuadros, Laura Ferreras Antolín. Hospital Dexeus Barcelona: Roser Porta. Hospital Donostia: Itziar Sota Busselo, Oihana Muga Zuriarrain. Hospital Fuenlabrada: Pilar Galán del Río.

Hospital General Castellón: Flavia Pronzato Cuello, Marta Dapena. Hospital General Catalunya: Laura Castells Vilella. Hospital Getafe: Beatriz Soto, Irene Cuadrado Pérez.

Hospital Gregorio Marañón: Elena Rincón López, Eva Dueñas, Jesús Saavedra, María del Mar Santos. Hospital Infanta Cristina Badajoz: Ana María Grande Tejada, Elena del Castillo Navío. Hospital Infanta Leonor: Adriana Navas, Beatriz Agundez Reigosa.

Hospital Infanta Sofía Madrid: Alfredo Tagarro García, Lorena Pérez Cid, María de la Serna Martínez, Rut del Valle. Hospital Jiménez Díaz: Ana Belén Jiménez.

Hospital Joan XXIII: Olga Calavia Garsaball. Hospital La Moraleja: Elvira Martín López, Jaime Carrasco. Hospital La Paz: Fernando Baquero, Luis Escosa, Paula Rodríguez-Molino, Teresa del Rosal. Hospital Las Palmas de Gran Canaria: Abian Montesdeoca Melián, Elena Colino Gil. Hospital Mataró. Consorci Sanitari del Maresme: Anna Vidal Moreso, Roser Díez Martín. Hospital Miguel Servet: Raquel Pinillos. Hospital Moncloa: Juana Barja Tur. Hospital Móstoles, Hospital Navarra: Alejandra Pérez García, Alexandra Álvarez de Blas, Felipe Garrido Martínez-Salazar, Laura Muñoz Saá.

Hospital Pontevedra: José Antonio Couceiro, Raquel Martínez Lorenzo. Hospital Príncipe de Asturias. Alcalá de Henares: José Beceiro. Hospital Puerta de Hierro: Fátima Ara, María Begoña Encinas Pardilla, Miguel Faustino Sánchez Mateos. Hospital Puerta del Mar: Almudena Alonso Ojembrana, Clotilde Fernández Gutiérrez del Álamo. Hospital Quirón BCN: Isabel Vives Oñós. Hospital Reina Sofía Córdoba: José Manuel Rumbao Aguirre. Hospital San Agustín de Avilés: Francisco Álvarez Breciano.

Hospital Sant Joan de Deu: Antoni Noguera Julian, Cláudia Fortuny Guasch, María Ríos Barnés. Hospital Severo Ochoa Leganés: Iciar Olabarrieta. Hospital Son Llatzer: Ana Filgueira Posse, Susana Herrero Pérez. Hospital Sureste Arganda: Marta Llorente.

Hospital Torrelodones: Isabel Llana Martín. Hospital Universitario de Canarias: Elena Colino Gil, Olga Alfonso Rodríguez. Hospital Vall d’Hebron: Beatriz Álvarez Vallejo, María Antoinette Frick, Pere Soler Palacín. Hospital Virgen Macarena Sevilla: Pedro Terol Barrero. Hospital de Melilla: María Montero Martín. Hospital Universitario Germans Trias i Pujol: Carlos Rodrigo, Clara Carreras Abad, Marta Nicolás López, Wilfredo Coroleu Lletget. Hospital de Basurto: Elisa Garrote Llanos, Henar Uriarte Gutiérrez, Joseba Iñaki Rementeria Radigales. Hospital Clínico Universitario Virgen de la Arrixaca: Ana Isabel Mensalvas Ruiz, Eloisa Cervantes. Hospital Juan Ramón Jiménez: Borja Croche Santander. Hospital Consorci Sanitari de Terrasa: Marina Fenoy, Vanessa Bonil Martínez. Hospital de Mérida: Mercedes García Reymundo, Virginia Vaquerizo Vaquerizo. Hospital Universitario Nuestra Señora de Candelaria: Beatriz Reyes Millán, Desireé Araceli Hernández, Dolores Romero Ramírez. Hospital Universitario Virgen del Rocío: Inés Marín Cruz, Lola Falcón. Hospital Josep Trueta: Borja Guach, Laia Solé. Hospital de Calella: Javier Cantero García. Hospital Rey Juan Carlos: Miguel Rico Pajares. Hospital Universitario de Torrejón: Carolina Guillamo, Katie Badillo Navarro. Hospital Río Hortega de Valladolid: Laura Sanz Rueda. Hospital Complejo Hospitalario de Navarra: Asier Oliver, María Malumbres. Hospital Complejo Hospitalario Universitario de Canarias: María Nieves González Bravo. Hospital Clínic de Barcelona: Ameth Hawkins, Anna Gonce, Dolors Salvia. Hospital Clínico de Valencia: David Navarri. Hospital Universitari de Vic: Montse Ruiz García.

Hospital Beatriz Ángelo: Sofía Costa Lima. Hospital Carlos Haya: Laura Ferreras Antolín.

Hospital San Pedro de Alcántara: María Jesús García García, Rebeca Martín. Hospital Alexandria University Children’s Hospital: Eman Hamza Hassan Hassan. Hospital Amsterdam UMC: Dasja Pajkrt. Hospital Azienda Ospedaliera Universitaria Federico II - Napoli: Francesco Raimondi, Serena Salomé. Hospital Barts Health: Eliza Alexander, Reeya Motha. Hospital Clinique St Jean Brussels: Valbona Selimaj. Hospital Bolton NHS Foundation Trust: Veronica Kennedy. Hospital Brasov Children’s Hospital: Falup Pecurariu Oana Gabriela. Hospital Bridgewater Community Healthcare: Ali Hilali.

Hospital Bukovinian State Medical University: Mykola Haras. Hospital Center for Clinical Epidemiology and Outcomes Research: Ioannis Kopsidas.

Hospital Charité University Medicine Berlín: Anne Sophie Schaper, Cornelia Fieterna-Sperling, Renate Krüger. Hospital Children’s Hospital Iceland: Valtyr Stefánsson. Hospital Children’s Health Ireland, Dublin: Bridget Freyne, Wendy Ferguson. Hospital Children’s Hospital Lucerne: Michael Büttcher. Hospital CHUV: Centre Hospitalier Universitaire Vaudois: Yves Fougere. Hospital Dokuz Eylül University Hospital: Hatice Karaoglu Asrak. Hospital Dr von Hauner Children’s Hospital: Anita Rack-Hock.

Hospital East Kent University Hospital: Foluso Thomas. Hospital Evelina London Children’s Hospital: Ira Shah, Julia Kenny, Jonathan Cohen, Tejshri Shah.

Hospital Faculty of Medicine Universitas Airlangga: Dominicus Husada. Hospital Fondazione IRCCS Policlinico S. Matteo: Angelini Micol, Francesca Garofoli, Giuseppina Lombardi. Hospital Fondazione Policlinico Universitario A. Gemelli IRCCS: Danilo Buonsenso. Hospital Great North Children’s Hospital: Eleri Williams. Hospital Great Ormond Street Children’s Hospital: Alasdair Bamford, Carina Elvegaard, Justin Penner, Kajal Bhagwandas, Rosie Crane, Seilesh Kadambari, Surangi Mendis, Waheeda Pagarkar, Xiang Li. Hospital Greatpoland Pediatric Center in Poznan: Ewelina Gowin.

Hospital Heraklion University Hospital: Eleni Vergadi. Hospital de São Francisco Xavier: Madalena Lopo Tuna. Hospital Tunku Azizah Kuala: Asrar Abu Bakar.

Hospital Universitário São João: Ana Reis Melo. Hospital Imperial College Healthcare: Bernie Borgstein, Ioannis Goniotakis, Wajanat Jan. Hospital Ippokrateio General Hospital: Charis Lampada, Emmanuel Roilides, Konstantina Charisi, Olga Tsiatsiou.

Hospital Irmandade da Santa Casa de Misericórdia de São Paulo: Camila Ohomoto de Morais. Hospital Kaplan Medical Center: Alex Guri. Hospital Karol Jonscher Clinical Hospital: Katarzyna Mazur-Melewska. Hospital Leicester Royal Infirmary Hospital: Srini Bandi. Hospital Leiden University Medical Center: Ann Vossen.

Hospital Leighton Hospital: Shailaja Kottapalli. Hospital Lewisham: Emma Gardiner, Lisa Capozzi. Hospital Manchester University NHS Foundation Trust: Adele Fitzgerald, Paddy McMaster, Sarah Holland. Hospital Norfolk and Norwich University Hospital NHS: Florence Waltson, Lucy Pocock, Myhill Jasmine, Samantha Scott. Hospital North Manchester General Hospital: Saskia Wills. Hospital Northern General Hospital: Benjamin Lindsey. Hospital Oxford University Hospital: Claire Douglas, Elizabeth David, Emily Lees, Kelly Dominic, Louise Pollard. Hospital Padeh-Poria Medical Center: Danny Glikman. Hospital Medical University of Bialystok: Artur Sulik, Dawid Lewandowski, Kacper Toczylowski. Hospital W.Bieganski Specialist Hospital: Adrianna Buciak, Ewa Majda-Stanislawska. Hospital Prof Hess Children’s Hospital: Petra Kaiser-Labusch. Hospital RHCYP: Laura Jones. Hospital Royal Alexandra Children’s Hospital: Katy Fidler. Hospital Royal Belfast Children’s Hospital: Sharon Christie. Hospital Royal Glasgow Children’s Hospital: Conor Doherty. Hospital Royal National ENT and Eastman Dental Hospital: Chrysa Spyridakou. Hospital Sheffield Children’s Hospital: Fiona Shackley, Samya Armoush. Hospital St George’s University Hospital: Laura Ferreras Antolín, Paul Heath, NgeeKeong Tan, Sana Ibrahim, Sarah Tizzard, Sharon Storey, Simon Drysdale, Simone Walter, Sinduja Janarthan. Hospital St Mary’s Hospital: Carolina Kachramanoglou, Helen Payne. Hospital St. Olavs University Hospital: Anastasios Smyrnaios. Hospital St. Orsola Hospital: Concetta Marisco, María Grazia Capretti. Hospital Sydney Children’s Hospitals Network: Gemma Héctor, Pamela Palasanthiran, Rachel Vernall. Hospital The Ruth Rappaport Children’s Hospital: Michal Meir. Hospital UK-Imperial College: Helen Payne, Hermione Lyall, Iro Mildred, Kailias Dhond, Karen McCarthy, Mona Almarzooqi. Hospital Cardiff and Vale UHB: Jennifer Evans, Jennifer Muller, Jessica Pitcher. Hospital Mid Cheshire: Joanne Tomlinson, Jones Amaryl, Shaila Kottapalli. Hospital Royal Sussex: Annalie Shears, Katy Fidler, Paul Frattaroli, Sonia Sobowiec, Vivien Richmond. Hospital Royal Cornwall: Kate Ralph, Kim Lindsey. Hospital Somerest Hospital: Christine Lanaghan, Jenny Langlands, Kirsty O’Brien, Rachel Crawley. Hospital St Thomas Hospital: Abirami Manian, Jonathan Cohen, Kent Alison. Hospital University Hospitals of Derby and Burton NHS Foundation Trust: Coral Smith, Melanie Hayman. Hospital University Hospitals Dorset: Francesca Díaz, Jemma Parratt, Nina Vanner. Hospital Walsall Healthcare NHS Trust: Ben Jones, Jessica Kane, Robert Chadwick. Hospital Universitair Ziekenhuis Brussel: Ina Foulon. Hospital Universitair Ziekenhuis Gent: Annelies Keymeulen.

Hospital University Children’s Hospital Zurich: Paolo Painoi, Shanya Sivakumaran, Victoria Parsonson. Hospital University College London Hospital: Camila Sen, Carolina Kachramanoglou, Christina Kortsalioudaki, Chrysa Spyridakou, Eleni Nastouli, Joanna Martín, Lucy Wellings, Sally Amor, Sarah Eisen. Hospital University General Hospital ATTIKON: Angeliki Tzaki, Artemis Mavridi, Chrysalena Loizou, Garyfallia Syridou, Sofía Karagiannidou, Vana Papaevangelou. Hospital University Hospital at Frankfurt: Horst Buxmann, Lujein Hafez, Malina Alexé, Thore Vogel. Hospital University Hospital Bulovka: Dita Smiskova, Jitka Bolchova. Hospital University Hospital Centre Hamburg-Eppendorf: Ulf Schulze-Sturm. Hospital University Hospital Crosshouse: Althaf Ansary.

Hospital University Hospital Luigi Sacco: Vania Giacomet. Hospital 12 de Octubre: Roberto Pedrero Tomé, Sara Vila Bedmar, Serena Villaverde González, Luis Prieto, Elisa Fernández Cooke, Jose Tomás Ramos Amador, Cristina Epalza, Cinta Moraleda, Adriana Shan, Jose Soler, Pablo Rojo, Daniel Blázquez-Gamero Hospital de Patras: Despoina Gkentzi and Sofia Benou.

## Supplementary Material


