# Supplementary material for: Prognostic Factors of Late-onset Hearing Loss in Infants With Congenital Cytomegalovirus and Normal Audiologic Assessment at Birth
Source: Pediatr Infect Dis J. 2025 Sep 9;45(1):1–10. doi: 10.1097/INF.0000000000004960 (PMC12688458; doi:10.1097/INF.0000000000004960)
Supplement: Supplementary file 1 [file inf-45-01-s001.pdf]

SUPPLEMENTAL DIGITAL CONTENT 1. Main clinical data of women with CMV infection in pregnancy

	level	No	Yes	p
Reason for cCMV diagnosis (%)	Maternal seroconversion during pregnancy	209 (38.8)	9 (28.1)	0,001
	Positive IgG e IgM with low avidity index (pregnancy)	42 (7.8)	1 (3.1)	.
	Antenatal fetal US abnormalities (pregnancy)	26 (4.8)	4 (12.5)	.
	Maternal symptomatic infection (pregnancy)	30 (5.6)	0 (0.0)	.
	Positive PCR in amniocentesis	25 (4.6)	1 (3.1)	.
	Failed newborn hearing screening test	6 (1.1)	0 (0.0)	.
	Clinical symptoms/signs consistent with cCMV at birth	63 (11.7)	14 (43.8)	.
	Hearing loss	1 (0.2)	0 (0.0)	.
	Neurologic abnormalities	17 (3.2)	1 (3.1)	.
	Prematurity	15 (2.8)	0 (0.0)	.
	Screening of baby born to mother with HIV	9 (1.7)	0 (0.0)	.
	Newborn universal screening program	18 (3.3)	0 (0.0)	.
	Other	78 (14.5)	2 (6.2)	.
Mother works with children (%)	Yes	36 (8.5)	5 (17.9)	0,186
	No	386 (91.5)	23 (82.1)	.
Children in daycare (%)	Yes	199 (38.6)	16 (45.7)	0,509
	No	317 (61.4)	19 (54.3)	.
Known contact with CMV (%)	Yes	24 (5.5)	3 (10.3)	0,497
	No	415 (94.5)	26 (89.7)	.
Mother with immunodeficiency (%)	Yes	20 (3.1)	0 (0.0)	0,442
	No	616 (96.9)	46 (100.0)	.
Amniocentesis performed (%)	Yes	167 (25.9)	9 (19.1)	0,391
	No	477 (74.1)	38 (80.9)	.
Weeks of gestation in first amniocentesis (median [IQR])		23.0 [21.0, 28.0]	24.0 [22.0, 25.0]	0,731
Cordocentesis performed (%)	Yes	11 (1.7)	0 (0.0)	0,787
	No	632 (98.3)	45 (100.0)	.
Weeks of gestation in first cordocentesis (median [IQR])		21.5 [20.2, 23.5]	NA [NA, NA]	NA
Cordocentesis: CMV PCR (fetal blood) (%)	Positive	8 (80.0)	0 (NaN)	NaN
	Negative	2 (20.0)	0 (NaN)	.
Cordocentesis: CMV Viral Load (median [IQR])		16452.0 [10629.0, 50031.0]	NA [NA, NA]	NA
Physical examination at birth (%)	Normal	526 (79.2)	25 (54.3)	<0.001
	Abnormal	138 (20.8)	21 (45.7)	.
Microcephaly (< -2 Z-Score or <= 2nd centile) (%)	Unchecked	634 (95.5)	39 (84.8)	0,005
	Checked	30 (4.5)	7 (15.2)	.
Seizures (%)	Unchecked	664 (100.0)	46 (100.0)	NaN
	Checked	0 (0.0)	0 (0.0)	.
Splenomegaly (%)	Unchecked	649 (97.7)	42 (91.3)	0,032
	Checked	15 (2.3)	4 (8.7)	.
Hepatomegaly (%)	Unchecked	645 (97.1)	40 (87.0)	0,001
	Checked	19 (2.9)	6 (13.0)	.
Hypotonia (%)	Unchecked	650 (97.9)	44 (95.7)	0,634
	Checked	14 (2.1)	2 (4.3)	.
Jaundice (%)	Unchecked	637 (95.9)	43 (93.5)	0,673
	Checked	27 (4.1)	3 (6.5)	.
Petechiae/ prupura (%)	Unchecked	624 (94.0)	38 (82.6)	0,008
	Checked	40 (6.0)	8 (17.4)	.
Small for gestational age (< -2 Z-Score or <= 2nd centile) (%)	Unchecked	609 (91.7)	36 (78.3)	0,005
	Checked	55 (8.3)	10 (21.7)	.
Congenital CMV diagnosis (%)	Fetal	113 (17.0)	9 (19.6)	0,904
	Newborn (first 21 days)	508 (76.6)	34 (73.9)	.
	Child (Retrospective diagnosis)	42 (6.3)	3 (6.5)	.
Treatment during pregnancy (%)	Yes	105 (24.4)	4 (20.0)	0,854
	No	325 (75.6)	16 (80.0)	.
Immunoglobulin (%)	Unchecked	595 (88.3)	44 (93.6)	0,381
	Checked	79 (11.7)	3 (6.4)	.
Valaciclovir (%)	Unchecked	644 (95.5)	46 (97.9)	0,699
	Checked	30 (4.5)	1 (2.1)	.
Type of treatment with immunoglobulin (%)	Preventive	34 (52.3)	0 (0.0)	0,46
	Treatment (confirmed fetal infection)	31 (47.7)	2 (100.0)	.
Gestational age (weeks) at first immunoglobulin dose (mean (SD))		25.1 (7.8)	26.0 (NA)	NA
mber of doses of immunoglobulin during pregnancy (mean (SD))		3.8 (3.2)	1.5 (0.7)	0,305
