# Supplementary material for: Prognostic Factors of Late-onset Hearing Loss in Infants With Congenital Cytomegalovirus and Normal Audiologic Assessment at Birth
Source: Pediatr Infect Dis J. 2025 Sep 9;45(1):1–10. doi: 10.1097/INF.0000000000004960 (PMC12688458; doi:10.1097/INF.0000000000004960)
Supplement: Supplementary file 2 [file inf-45-01-s002.pdf]

SUPPLEMENTAL DIGITAL CONTENT 2. Time series analysis about the development of LO-SNHL

Time of follow-up (months)	n	survival	std.err	lower 95% CI	upper 95% CI
3.57	45	0.978	0.022	0.936	1.000
4.07	44	0.956	0.031	0.897	1.000
6.21	43	0.933	0.037	0.863	1.000
7.64	42	0.911	0.042	0.832	0.998
8.11	41	0.889	0.047	0.802	0.986
8.96	40	0.867	0.051	0.773	0.972
10.43	39	0.844	0.054	0.745	0.957
10.54	38	0.822	0.057	0.718	0.942
11.61	37	0.800	0.060	0.691	0.926
12.18	36	0.756	0.064	0.640	0.892
14.11	34	0.733	0.066	0.615	0.875
15.14	33	0.711	0.068	0.590	0.857
15.18	32	0.689	0.069	0.566	0.838
15.96	31	0.667	0.070	0.542	0.820
16.18	30	0.644	0.071	0.519	0.801
17.25	29	0.622	0.072	0.496	0.781
18.68	28	0.600	0.073	0.473	0.762
25.64	27	0.578	0.074	0.450	0.742
25.68	26	0.556	0.074	0.428	0.721
25.71	25	0.533	0.074	0.406	0.701
26.18	24	0.511	0.075	0.384	0.680
27.04	23	0.489	0.075	0.363	0.659
34.32	22	0.467	0.074	0.342	0.638
37.71	21	0.444	0.074	0.321	0.616
38.82	20	0.422	0.074	0.300	0.594
39.25	19	0.400	0.073	0.280	0.572
39.29	18	0.378	0.072	0.260	0.550
39.43	17	0.356	0.071	0.240	0.527
40.14	16	0.333	0.070	0.221	0.504
40.93	15	0.311	0.069	0.201	0.481
41.14	14	0.289	0.068	0.183	0.457
43.21	13	0.267	0.066	0.164	0.433
45.50	12	0.244	0.064	0.146	0.409
48.71	11	0.222	0.062	0.129	0.384
50.29	10	0.200	0.060	0.112	0.359
51.75	9	0.178	0.057	0.095	0.333
51.86	8	0.156	0.054	0.079	0.307
52.11	7	0.133	0.051	0.063	0.281
52.50	6	0.111	0.047	0.049	0.254
54.54	5	0.089	0.042	0.035	0.227
58.11	4	0.067	0.037	0.022	0.199
59.11	3	0.044	0.031	0.012	0.172
65.61	2	0.022	0.022	0.003	0.154
65.71	1	0.000	-	-	-
